# Early Local Treatment Delays Multiple Metastases in Non‐Small Cell Lung Cancer With Solitary Distant Metastasis: A Multicenter Retrospective Cohort Study

**DOI:** 10.1002/mco2.70904

**Published:** 2026-09-02

**Authors:** Kang Miao, Siyu Lei, Xiaobin Zheng, Shanshan Yang, Yujing Li, Sihui Chen, Qian Miao, Longfeng Zhang, Zihua Zou, Haipeng Xu, Gen Lin

**Affiliations:** ^1^ Department of Thoracic Oncology NHC Key Laboratory of Cancer and Metabolism Clinical Oncology School of Fujian Medical University, Fujian Cancer Hospital Fuzhou China; ^2^ Department of Medical Oncology Beijing Tuberculosis and Thoracic Tumor Research Institute Beijing Chest Hospital, Capital Medical University Beijing China

**Keywords:** local treatment, non‐small cell lung cancer, restricted mean survival time, solitary distant metastasis, time to multiple metastases

## Abstract

Solitary distant metastasis (SDM) is a distinct oligometastatic subtype associated with a relatively favorable prognosis. This multicenter retrospective study including 139 non‐small cell lung cancer (NSCLC) patients with SDM (2019–2024) was conducted to investigate prognostic factors for multiple metastases and identify the critical time window for local treatment. Statistical analyses included Cox regression, Kaplan–Meier survival analysis, and restricted mean survival time (RMST). Results showed that driver gene status, tumor staging, systemic therapy, and local treatment were significantly associated with progression‐free survival (PFS). Notably, only early local treatment significantly delayed the onset of multiple metastases (hazard ratio [HR] = 0.55, 95% confidence interval [CI]: 0.30–0.99, *p* = 0.05), prolonging the median time to multiple metastases by nearly 14 months (36.4 vs. 22.6 months). Kaplan–Meier and RMST analyses revealed no distinct plateau phase, indicating the absence of an absolute temporal cutoff between “true oligo‐metastasis” and “transitional metastasis,” which suggests that SDM in advanced NSCLC is an inherently indolent disease state. Using the maximum selected rank statistics method, an optimal cutoff of 138 days was identified to distinguish early from delayed local treatment, which requires further prospective validation.

## Graphical abstract

In NSCLC patients with solitary distant metastasis (SDM), early local treatment significantly delays the progression to multi‐metastasis by 14 months (HR = 0.55) compared to delayed or no treatment. This multicenter cohort study highlights SDM as an indolent state, emphasizing the critical time window for effective local intervention.

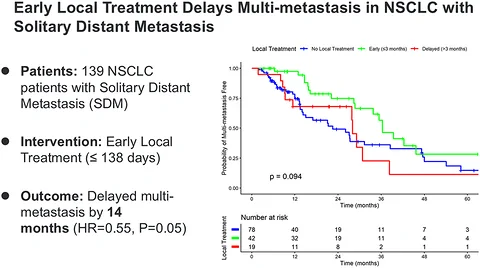

## 1 Introduction

Lung cancer oligo‐metastasis represents a distinct biological state characterized by limited metastatic burden and localized distribution, intermediated between localized primary tumors and widespread systemic metastases [1]. Guidelines from the American Society for Radiation Oncology (ASTRO) and the European Society for Radiotherapy and Oncology (ESTRO) define it as the presence of up to five metastatic lesions involving a maximum of three organs [2]. Oligo‐metastatic patients account for approximately 10%‐15% of the metastatic non‐small cell lung cancer (NSCLC) population, but there is significant survival variability within this subgroup, which may be related to differences in tumor biological behavior and treatment strategies [3].

Clinical management of oligo‐metastasis remains controversial, especially regarding the role of local treatment. Previous studies have shown that local treatment (e.g., surgery, radiotherapy) can improve survival in oligo‐metastatic NSCLC, but the optimal timing and modality of local treatment are not well established. Moreover, the lack of a unified definition and prognostic stratification criteria for oligo‐metastasis has led to inconsistent treatment decisions in clinical practice, resulting in heterogeneous outcomes.

“True oligo‐metastasis” refers to a condition characterized by limited number of metastatic lesions, relatively indolent biological behavior, and a low metastatic potential toward developing multiple metastases, which may achieve long‐term control or even complete cure through local treatments [4]. “Transitional metastasis” refers to a transitional state rapidly progressing toward systemic multiple metastases, despite the presence of only a small number of metastatic lesions on imaging [5]. Clinically, it remains a challenge in effectively distinguish between “true oligo‐metastasis” and “transitional metastasis.” Current predictive methods relying on circulating tumor cells (CTCs) or circulating tumor DNA (ctDNA) are limited by cost and accessibility [6]. Although such molecular markers can reflect tumor burden, in real‐world clinical settings, these molecular monitoring technologies are limited by detection costs and the feasibility of high‐frequency dynamic monitoring, making it difficult to achieve standardization and widespread adoption [7]. In light of this, survival analysis methods based on the time dimension show unique advantages, as the time trajectory of disease progression inherently reflects the biological behavior of the tumor. If the survival curve exhibits a significant “plateau phase,” it may be possible to achieve efficient stratification of metastatic states by setting a time threshold.

In clinical practice, the feasibility of local treatment diminishes as the number of metastatic lesion increases [8]. When the number of metastatic lesions exceeds one, both the technical difficulty and treatment risk of multisite interventions rise exponentially [9]. Among them, solitary distant metastasis (SDM), as a special subtype of oligo‐metastasis, specifically refers to a single metastatic lesion in a single organ (stage M1b lung cancer) [10]. This anatomical feature provides ideal conditions for local radical interventions (such as surgical resection and stereotactic radiotherapy), which can not only reduce complications caused by multisite interventions but also delay disease progression through precise local control [11]. Owing to its unique biological behavior and anatomical characteristics, this subtype has become a precise intervention target with significant clinical value for local treatment among metastatic tumors [12]. However, few studies have focused on SDM alone, and the optimal time window for local treatment and the existence of a temporal threshold to distinguish “true oligo‐metastasis” from “transitional metastasis” remain unclear.

Therefore, this study aims to (1) identify prognostic factors driving the progression from SDM to multiple metastases, with a focus on local treatment modalities and timing and (2) investigate whether a critical time point can be identified in a single time dimension to distinguish long‐term stable “true oligo‐metastasis” from short‐term “transitional metastasis.”

## 2 Results

### 2.1 Cohort Characteristics and Follow‐Up

**FIGURE 1:**
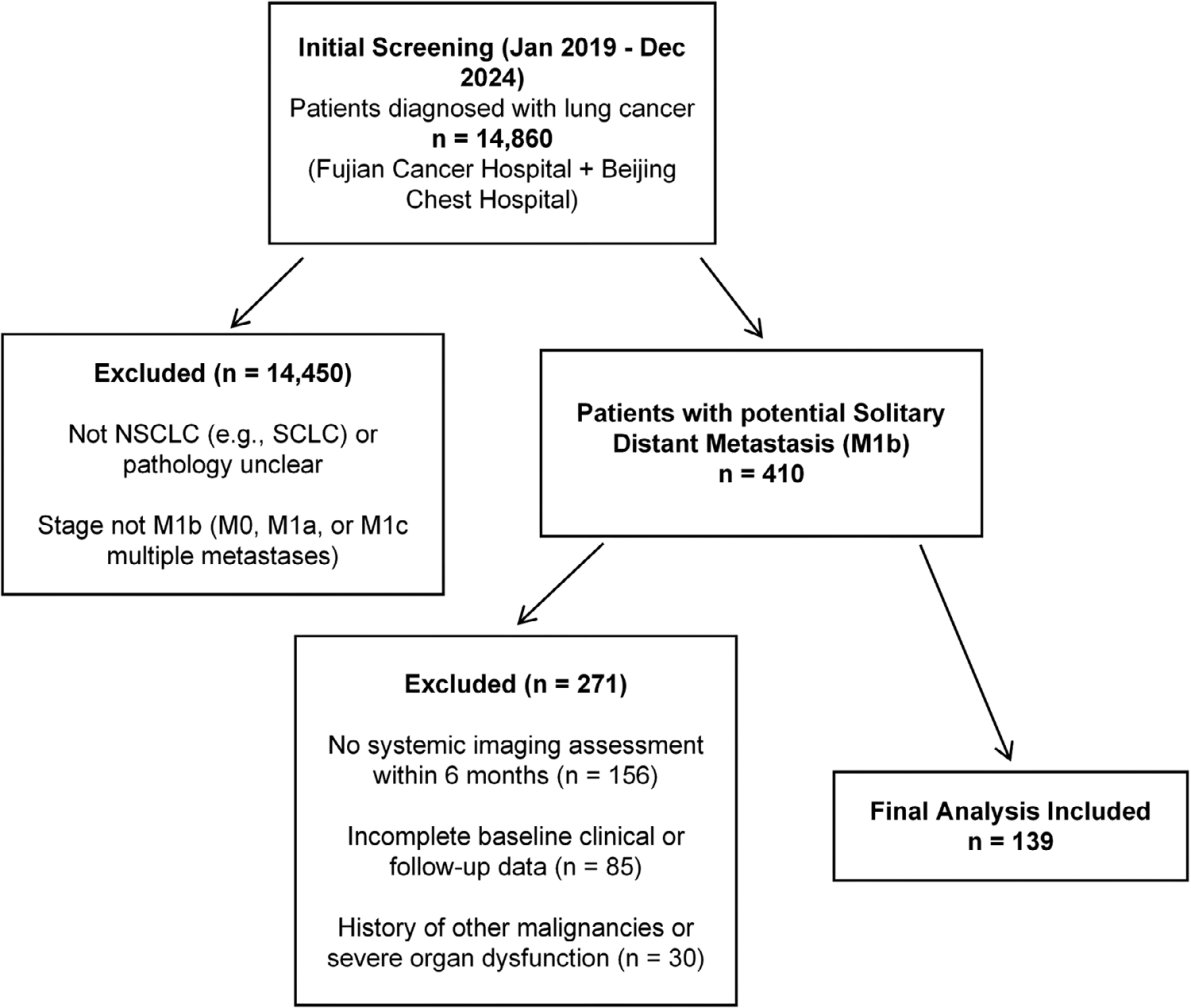
STROBE flowchart of patient selection, enrollment, and screening process for identifying eligible patients with solitary distant metastasis (SDM) in non‐small cell lung cancer (NSCLC).

The patient selection process is illustrated in Figure 1. A total of 14,860 lung cancer patients were ultimately screened, and 139 eligible patients with SDM were finally included in the analysis. Baseline characteristics are summarized in Table 1. All 139 included patients had complete baseline and follow‐up data, with no missing values and no losses to follow‐up during the observation period.

**TABLE 1:** Baseline characteristics of the study cohort.

Characteristics	Category	*n*	%
**Gender**	Male	89	64.0
	Female	50	36.0
**Age group**	≤60	61	43.9
	>60	78	56.1
**Smoking history**	None	80	57.6
	Yes	28	20.1
	Quit	31	22.3
**ECOG score**	0–1	129	92.8
	2–4	10	7.2
**Pathological type**	Adenocarcinoma	107	77.0
	Squamous cell carcinoma	27	19.4
	Other	5	3.6
**Driver gene**	None	61	43.9
	EGFR	48	34.5
	ALK	9	6.5
	Other	21	15.1
**Stage T**	1–2	79	56.8
	3–4	60	43.2
**Stage N**	0–1	50	36.0
	2–3	89	64.0
**Oligo‐metastatic site**	Brain	47	33.8
	Bone	71	51.1
	Other	21	15.1
**Systemic treatment**	Targeted therapy	65	46.8
	Chemotherapy	70	50.4
	Other	4	2.9
**Local treatment**	None	78	56.1
	Surgery	9	6.5
	Radiotherapy	47	33.8
	Other	5	3.6
**Local treatment site**	None	78	56.1
	Primary tumor	15	10.8
	Metastatic tumor	44	31.7
	Both	2	1.4
**Timing of local treatment**	No local treatment	78	56.1
	Within 3 months	42	30.2
	Over 3 months	19	13.7

The median follow‐up time for the entire cohort was 18 months. By the data cutoff date, 68 patients (48.9%) had developed multiple metastases. Among the 139 patients, 61 patients (43.9%) received local therapy, mostly radiotherapy (47 cases, 77.0%), targeting primary or metastatic lesions, with 68.9% of these patients receiving treatment within 3 months. The majority of patients had good performance status (ECOG 0–1, 92.8%). Bone (51.1%) and brain (33.8%) were the most common metastatic sites. Histologically, adenocarcinoma was the predominant histologic subtype (107 cases, 77.0%), followed by squamous cell carcinoma (27 cases, 19.4%), with five cases of unclassified NSCLC. Systemic therapy included targeted therapy (65 cases, 46.8%) and chemotherapy (70 cases, 50.4%).

### 2.2 Prognostic Factors for Progression‐Free Survival (PFS)

Univariate Cox regression analysis for PFS is shown in Table 2. The results showed that driver gene status, tumor stage, systemic therapy, and local treatment strategies were all significantly associated with PFS. Patients with EGFR mutations (hazard ratio [HR] = 0.547, *p* = 0.008) and other positive driver gene (HR = 0.520, *p* = 0.025) had significantly reduced progression risks. Conversely, advanced primary tumor stage (T3–4) (HR = 1.551, *p* = 0.033) and lymph node metastasis (N2–3) (HR = 1.578, *p* = 0.037) predicted rapid tumor progression.

**TABLE 2:** Univariate cox regression analysis for PFS.

		*p*	HR
**Gender**	Male	0.173	Reference
	Female		0.751 (0.498–1.134)
**Age group**	≤60	0.721	Reference
	>60		1.075 (0.722–1.602)
**Smoking history**	None	0.177	Reference
	Yes	0.063	1.703 (0.971–2.988)
	Quit	0.758	1.086 (0.642–1.839)
**ECOG score**	0–1	0.971	Reference
	2–3		1.015 (0.443–2.329)
**Pathological type**	Adenocarcinoma	0.225	Reference
	Squamous cell carcinoma		1.403 (0.812–2.422)
**Driver gene**	None	0.013	Reference
	EGFR	0.008	0.547 (0.350–0.854)
	Other	0.025	0.520 (0.294–0.923)
**Stage T**	T1–2	0.033	Reference
	T3–4		1.551 (1.037–2.320)
**Stage N**	N0–1	0.037	Reference
	N2–3		1.578 (1.028–2.422)
**Oligo‐metastatic site**	Brain	0.823	Reference
	Bone	0.534	0.872 (0.566–1.343)
	Other	0.774	0.910 (0.479–1.731)
**Systemic treatment**	Targeted therapy	0.009	Reference
	Chemotherapy		1.741 (1.151–2.633)
**Local treatment**	None	0.017	Reference
	Surgery	0.035	0.373 (0.149–0.932)
	Radiotherapy	0.021	0.601 (0.390–0.927)
	Other	0.112	0.201 (0.028–1.454)
**Local treatment site**	None	0.017	Reference
	Primary tumor	0.016	0.401 (0.191–0.842)
	Metastatic tumor	0.031	0.612 (0.392–0.956)
	Both	0.138	0.223 (0.031–1.623)
**Timing of local treatment **	None	0.005	Reference
	Within 3 months	0.001	0.447 (0.276–0.723)
	Over 3 months	0.352	0.762 (0.430–1.351)

Regarding treatment, patients receiving targeted therapy had a 42.5% lower progression risk compared with the chemotherapy group (HR = 0.575, *p* = 0.009). For local therapy, both surgery (HR = 0.373, *p* = 0.035) and radiotherapy (HR = 0.601, *p* = 0.021) were associated with significantly improved PFS, regardless of whether treatment was directed at the primary tumor (HR = 0.401, *p* = 0.016) or metastatic lesions (HR = 0.612, *p* = 0.031). Notably, patients receiving early local treatment (≤3 months) achieved a 55.3% reduction in progression risk (HR = 0.447, *p* = 0.001) compared with those without local treatment, corresponding to a median PFS prolongation of 18 months. No significant PFS benefit was observed in the delayed treatment group compared with the untreated group (*p* = 0.352), highlighting a distinct time window effect.

### 2.3 Factors Associated With Progression to Multiple Metastases

**FIGURE 2:**
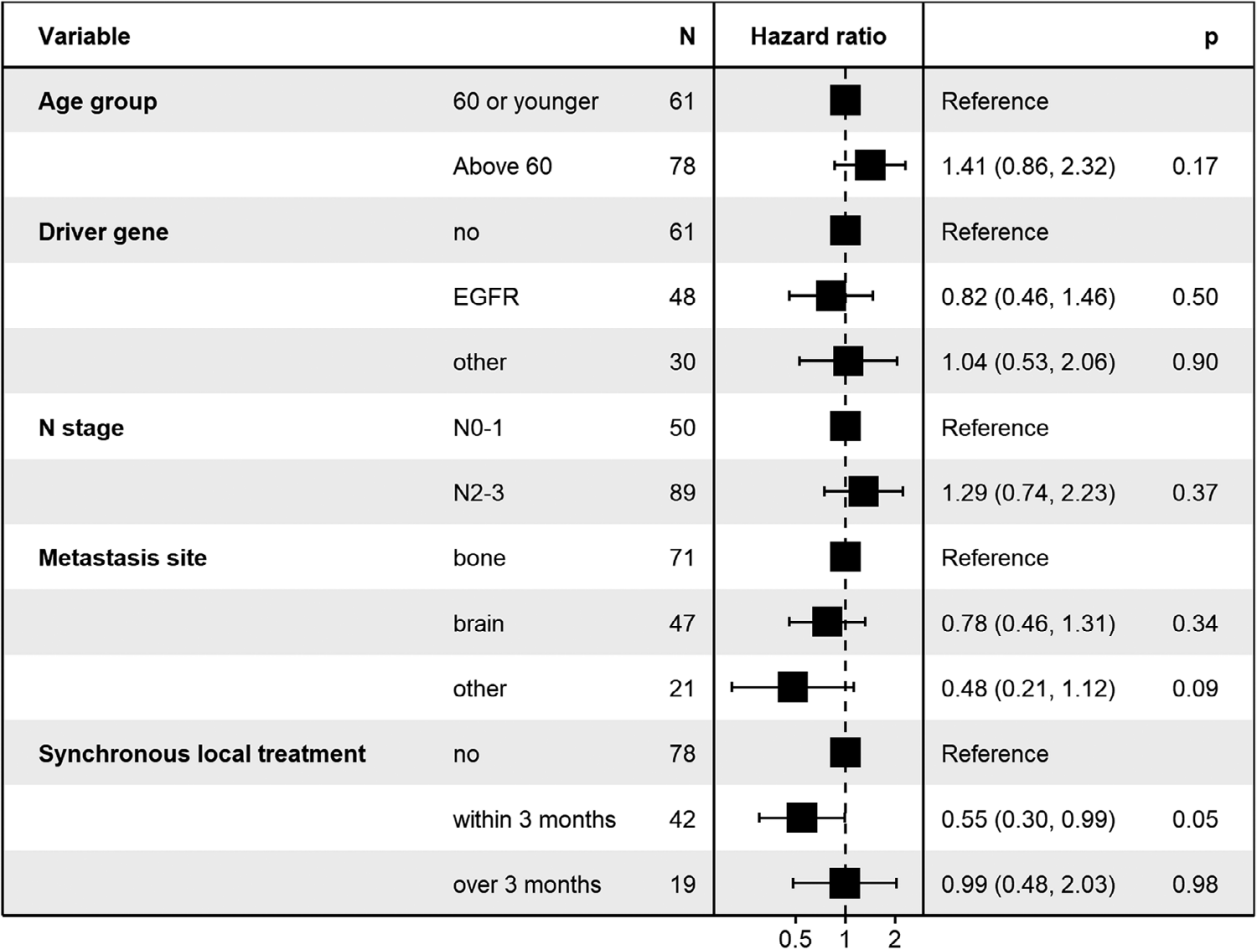
Multivariate Cox proportional hazards regression analysis of factors associated with multiple metastases. Hazard ratios (HR) and 95% confidence intervals (CI) for varying clinical factors. The vertical dashed line at HR = 1 indicates no association with multiple metastases risk.

We further investigated risk factors specifically for the development of multiple metastases (Table 3). Univariate Cox regression analysis of prognostic factors for multiple metastases identified age group (*p* = 0.116), N stage (*p* = 0.187), and local treatment timing (*p* = 0.101) as variables meeting the inclusion threshold of *p* < 0.2. Considering their established clinical significance, driver genes and metastatic sites were also entered into multivariate Cox regression. In the multivariate Cox regression analysis (Figure 2), early local treatment (≤3 months) emerged as the sole independent protective factor against multiple metastases (HR = 0.55, 95% confidence interval [CI]: 0.30–0.99, *p* = 0.05). Other variables, including age, driver gene status, N stage, and metastatic site, did not reach statistical significance.

**TABLE 3:** Univariate cox regression analysis for prognostic factors associated with multiple metastases.

		*p*	HR
**Gender**	Male	0.325	Reference
	Female		0.782 (0.479–1.277)
**Age group**	≤60	0.116	Reference
	>60		1.475 (0.908–2.394)
**Smoking history**	None	0.498	Reference
	Yes	0.567	1.252 (0.580–2.703)
	Quit	0.355	0.714 (0.349–1.458)
**ECOG score**	0–1	0.551	Reference
	2–3		1.518 (1.126–2.124)
**Pathological type**	Adenocarcinoma	0.725	Reference
	Squamous cell carcinoma		0.858 (0.364–2.018)
**Driver gene**	None	0.872	Reference
	EGFR	0.667	0.884 (0.504–1.551)
	Other	0.969	1.013 (0.518–1.981)
**Stage T**	T1–2	0.607	Reference
	T3–4		1.139 (0.694–1.870)
**Stage N**	N0–1	0.187	Reference
	N2–3		1.410 (0.846–2.349)
**Oligo‐metastatic site**	Brain	0.549	Reference
	Bone	0.516	1.185 (0.710–1.977)
	Other	0.557	0.777 (0.334–1.804)
**Systemic treatment**	Targeted therapy	0.495	Reference
	Chemotherapy		1.191 (0.721–1.969)
**Local treatment**	None	0.393	Reference
	Surgery	0.609	0.799 (0.337–1.892)
	Radiotherapy	0.108	0.648 (0.382–1.099)
	Other	0.440	0.456 (0.062–3.342)
**Local treatment site**	None	0.343	Reference
	Primary tumor	0.141	0.495 (0.195–1.261)
	Metastatic tumor	0.248	0.735 (0.437–1.238)
	Both	0.425	0.445 (0.061–3.258)
**Timing of local treatment **	None	0.094	Reference
	Within 3 months	0.039	0.552 (0.314–0.970)
	Over 3 months	0.991	0.996 (0.507–1.957)

**FIGURE 3:**
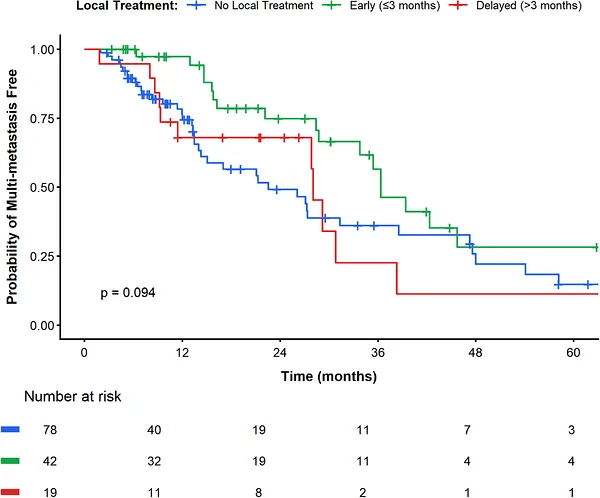
Time to multiple metastases by local treatment timing. Comparisons of multiple metastases‐free survival probabilities among patients receiving early local treatment (≤3 months, green), delayed local treatment (>3 months, red), and no local treatment (blue).

Kaplan–Meier curves for time to multiple metastases by local treatment timing (within 3 months vs. over 3 months vs. no treatment) are presented in Figure 3 and corroborate these findings. The median time to multiple metastases was 36.4 months for the early local treatment group, compared with 28.0 months for the delayed group and 22.6 months for the untreated group.

### 2.4 Subgroup Analysis: Surgery Versus Radiotherapy

To determine the optimal modality for local control, we conducted a subgroup analysis on the 61 patients who received local treatment. All local treatments were administered on the basis of standard systemic therapy, including targeted therapy or chemotherapy. Of these, 9 underwent surgical resection, 47 received radiotherapy, and 5 received combined/other modalities. Kaplan–Meier analysis revealed comparable outcomes between the two major modalities: the median time to multiple metastases was 33.8 months in the surgical group and 35.4 months in the radiotherapy group. Multivariate Cox regression confirmed no significant difference in the risk of multiple metastases between surgery and radiotherapy (HR = 1.21, 95% CI: 0.48–3.02, *p* = 0.689) (Table S1).

### 2.5 Time Threshold Exploration

**FIGURE 4:**
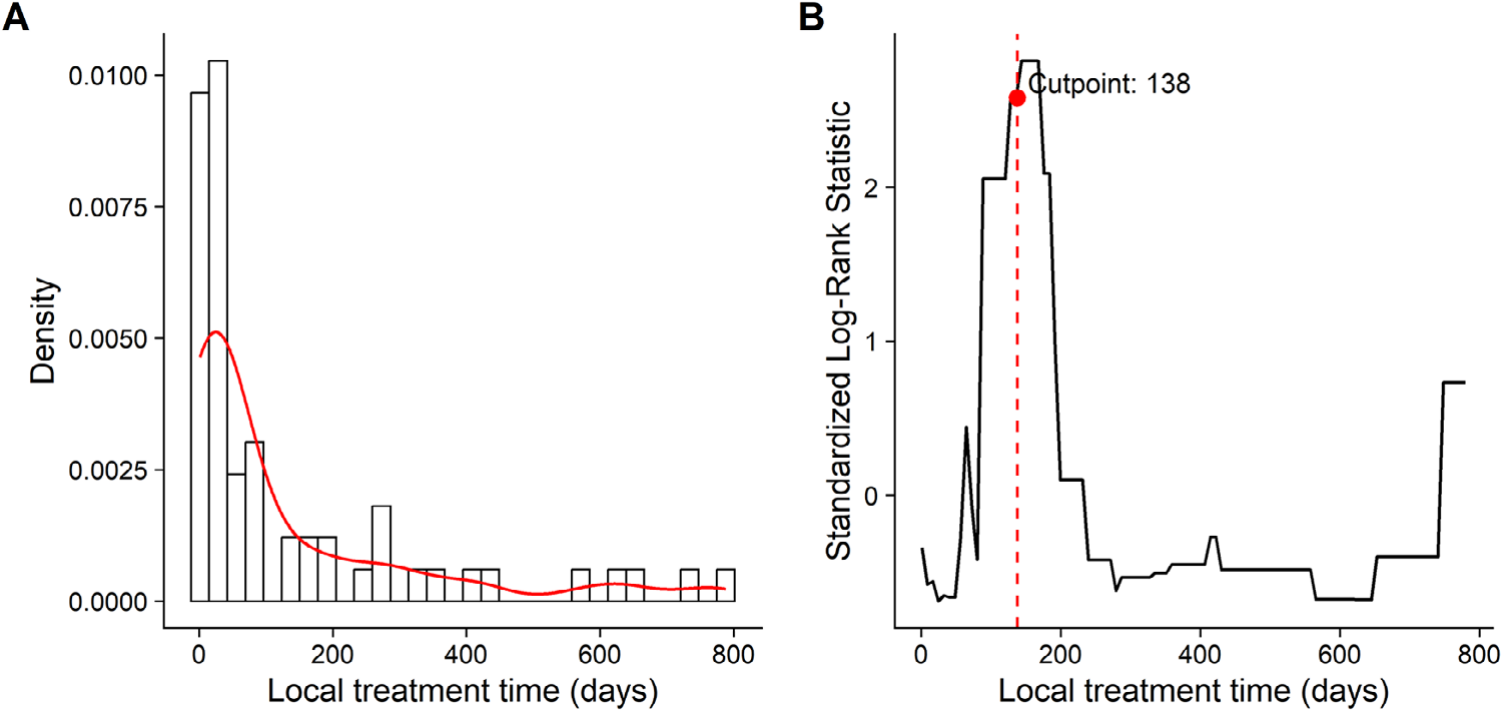
Distribution characteristics and optimal cut‐off point analysis of local treatment timing. (A) Histogram and kernel density estimation curve illustrating the frequency density of local treatment timing. (B) Maximum selected rank statistics analysis identifying the optimal temporal cut‐off point for multiple metastases‐free survival.

In clinical research and practice involving lung cancer oligo‐metastasis, a 3‐month interval is commonly adopted as an empirical cutoff for “early local treatment.” To address the subjective limitations of the “empirical 3‐month” threshold, we explored the optimal cutoff point for local treatment timing. The distribution of local treatment timing among the 61 treated patients is visualized in Figure 4A. The frequency distribution revealed that the timing of local treatment predominantly peaked within the 30–60 day window, showing an overall right‐skewed distribution, which aligned with the clinical practice of initiating local consolidative therapy promptly after diagnosis. Using the maximum selected rank statistics (MSRS) method, we identified 138 days (≈4.6 months) as the optimal cutoff point that achieved the best stratification of multiple metastasis‐free survival (Figure 4B).

**FIGURE 5:**
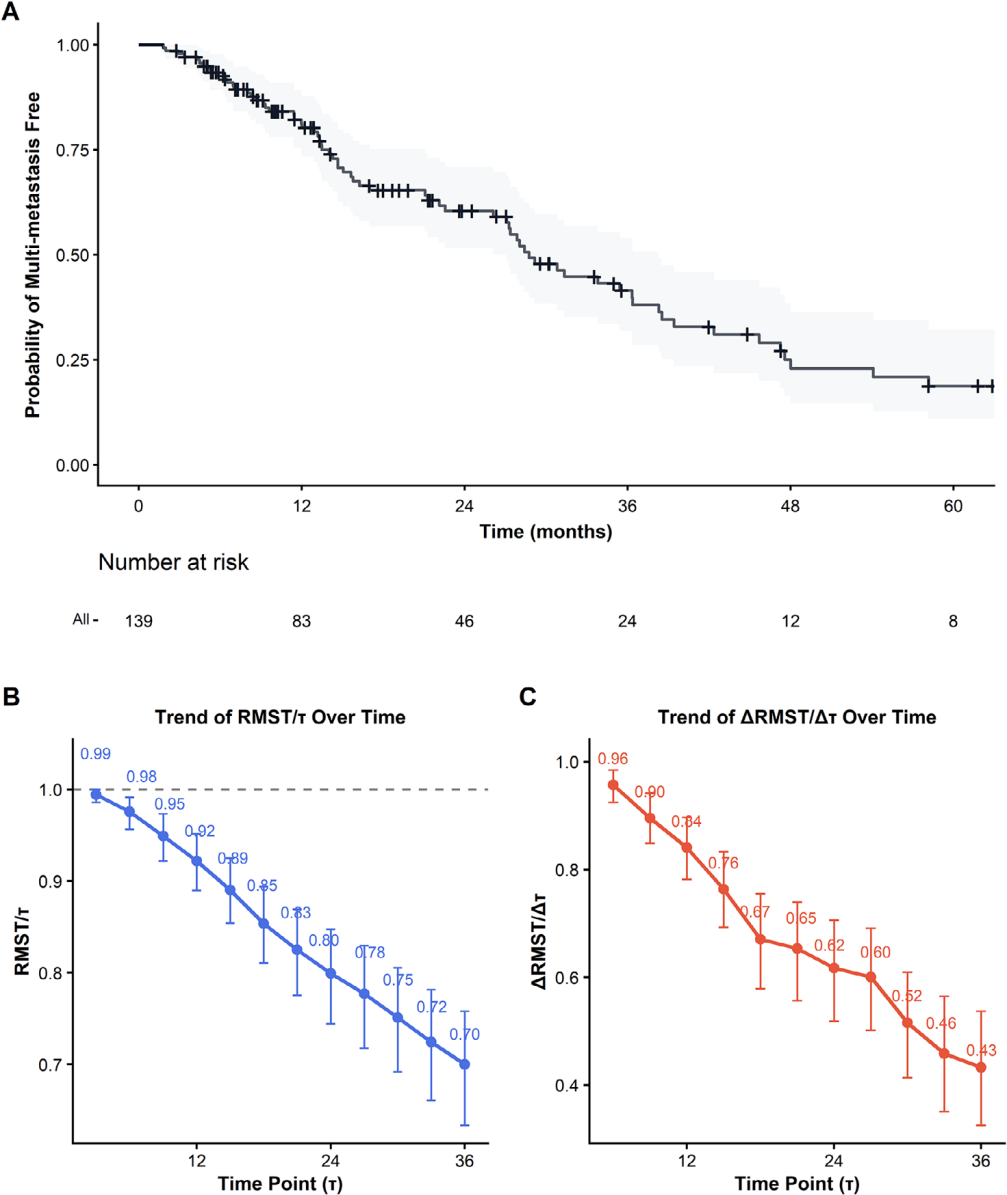
Multiple metastases‐free survival curve and restricted mean survival time (RMST) analysis. (A) Overall Kaplan–Meier curve for multiple metastases‐free survival in the study cohort. (B) Trend of the RMST/*τ* ratio over the observation time (*τ*). (C) Trend of the rate of change in RMST (ΔRMST/Δ*τ*) over time.

To define the temporal cutoff point between “true oligo‐metastasis” and “transitional metastasis,” we analyzed the Kaplan–Meier survival curve in conjunction with RMST‐derived indicators. The Kaplan–Meier curve (Figure 5A) showed that the probability of multiple metastases‐free survival declined continuously over follow‐up, with a 24‐month cumulative survival rate of 0.605 (95% CI: 0.516–0.709). In addition, no distinct plateau phase was observed. Consistent with this, the RMST/*τ* ratio decreased dynamically, dropping from 0.99 at *τ* = 3 months to 0.70 at *τ* = 36 months (Figure 5B), and the rate of change (ΔRMST/Δ*τ*) also showed a steady downward trend (Figure 5C). These findings confirm that no absolute temporal cutoff can reliably distinguish “true oligo‐metastasis” from “transitional metastasis” in this cohort, which suggests that the risk of subsequent multiple metastases persisted continuously over time, rather than being confined to a predefined early time window.

## 3 Discussion

Our study confirmed that early local treatment of SDM (M1b) was an independent protective factor against multiple metastases (HR = 0.55, *p* < 0.05), prolonging median time to multiple metastases by 14 months relative to the untreated group (36.4 vs. 22.6 months). This aligns with the “early intervention” theory in oligo‐metastasis, where prompt eradication of dominant metastatic lesions may reduce CTC dissemination and inhibit micro‐metastasis clonal evolution [13].

A key finding of this study is the identification of an optimal cutoff threshold of 138 days (4.6 months). In the SABR‐COMET trial, early local treatment in PS 0–1 patients improved 5‐year OS from 17.7% to 42.3% (HR = 0.47, *p* = 0.006) [14]. While it utilized a 3‐month empirical cutoff, our data‐driven analysis suggests that the effective window for local intervention may be wider than previously thought.

The optimal cutoff of 138 days (4.6 months) identified in this study is slightly longer than the clinically empirical 3‐month threshold. This difference may be attributed to real‐world factors such as patient selection patterns, treatment accessibility, and preoperative evaluation time. The 3‐month threshold is widely used due to its simplicity and clinical convenience, but it is subjective and lacks robust data‐driven validation. Our data‐driven 138‐day cutoff provides an exploratory reference, which suggests that the “time window” for effective local treatment may be broader than the empirical 3‐month threshold. However, given the sample size constraints and the intrinsic biological heterogeneity across different metastatic sites, this threshold should be interpreted as a hypothesis‐generating reference rather than a definitive clinical guideline. Clinical decision‐making must rigorously account for individual patient differences and organ‐specific biological behaviors.

Analysis of factors influencing PFS revealed that patients harboring actionable driver mutations such as EGFR or ALK had significantly prolonged PFS, which was directly attributable to the widespread application of targeted therapy. However, EGFR and ALK alterations are well‐established adverse prognostic factors for the development of multiple distant metastases [15]. Biologically, these genetic alterations can promote tumor cell migration and angiogenesis by activating pathways like RAS‐MAPK and PI3K‐AKT, thereby enhancing the metastatic potential of tumors [16]. In further exploration of risk factors affecting multiple metastases, we observed that neither driver mutation status nor systemic treatment modalities exerted a significant influence on multiple metastases. This result suggested that although targeted therapy can prolong PFS by inhibiting the proliferation of dominant lesions and partially counteracts the enhanced biological aggressiveness conferred by driver genes, its efficacy in eradicating existing micro‐metastases and halting their latent clonal outgrowth remains limited.

We also addressed the debate regarding local treatment modalities. Our subgroup analysis showed no significant difference in the risk of subsequent multiple metastases between surgery and radiotherapy. This aligns with recent consensus that the choice of modality should be driven by tumor location, patient surgical eligibility, and institutional expertise rather than presumed differences in oncologic efficacy.

Our analysis of RMST and survival kinetics revealed no distinct plateau phase in the multiple metastasis‐free survival curve, challenging the binary classification of “true” versus “transitional” oligometastasis based solely on time. Instead, the continuous risk of progression suggests that SDM in advanced NSCLC is an inherently indolent disease state rather than a distinct, curable entity or a fleeting transitional phase. This implies that while durable cure remains uncommon, long‐term disease control is achievable through timely multimodal therapy.

This study is limited by its retrospective design and relatively modest sample size, which may introduce selection bias (e.g., the choice of local treatment may be influenced by physician experience and patient characteristics [17]). Detailed power analysis utilizing R software based on the Schoenfeld formula (two‐sided *α* = 0.05) revealed an inherent limitation. While our sample size (*n* = 139, 68 events) provides sufficient prespecified power (87.07%) to detect a clinically established effect size (HR = 0.47, derived from the landmark Phase II SABR‐COMET trial), the actual post‐hoc power based on our observed in‐cohort effect size (HR = 0.55) is 68.67%, falling short of the conventional 80% threshold. Although we adjusted for potential confounders through multivariate Cox regression, unmeasured factors (such as tumor mutational burden and ctDNA levels) may still affect the results. Furthermore, intrinsic biological heterogeneity across different metastatic sites (51.08% bone vs. 33.8% brain) was observed, which directly constrains the generalizability of our overall pooled findings. Despite adjustment for established confounders, organ‐specific subgroup analyses were not feasible due to sample size constraints. Thus, large‐scale prospective studies are critically warranted to validate the findings and explore organ‐specific treatment strategies for SDM.

## 4 Conclusions

While early local treatment was associated with delayed onset of multiple metastases and our data suggest SDM behaves as an inherently indolent disease state, the 138‐day threshold serves as an exploratory reference requiring further prospective validation. Given the limited sample size and inherent organ heterogeneity, large‐scale, organ‐specific prospective validation studies are warranted to corroborate these findings and guide clinical practice.

## 5 Methods

### 5.1 Data Source and Study Design

This multicenter retrospective cohort study enrolled NSCLC patients with SDM from the Department of Thoracic Oncology, Fujian Cancer Hospital, and the Department of Oncology, Beijing Chest Hospital, between January 2019 and December 2024. The study was approved by institutional ethics committees and conducted in accordance with the Declaration of Helsinki; all patient data were anonymized prior to analysis. To minimize selection bias and maximize statistical power, all consecutive patients meeting the eligibility criteria during the study period were included.

### 5.2 Inclusion Criteria

1. Pathologically confirmed NSCLC (adenocarcinoma, squamous cell carcinoma, or other non‐small cell types);
2. Single‐organ solitary metastasis (M1b stage) confirmed by CT/MRI/PET‐CT;
3. At least one whole‐body systemic imaging evaluation was performed within 6 months post‐SDM diagnosis;
4. Age ≥ 18 years with complete baseline clinical and follow‐up data.

### 5.3 Exclusion Criteria

1. Prior multiple metastases before SDM diagnosis (i.e., converted from multi‐metastatic to oligo‐metastatic status through treatment);
2. Severe cardiopulmonary/hepatic/renal dysfunction or concurrent active secondary malignancies.

### 5.4 Data Collection

1. Baseline characteristics: Sex, age (stratified at 60 years), smoking history (current/former/never), ECOG performance status (0–1 vs. 2–4).
2. Tumor characteristics: Histology (adenocarcinoma/squamous cell carcinoma/other), driver gene status (e.g., EGFR 19del/L858R, ALK fusion), primary tumor TNM stage (T1–2 vs. T3–4; N0–1 vs. N2–3), metastatic organ (brain/bone/other).
3. Treatment information: Local therapy type (surgical resection/radiotherapy), timing (within 3 months vs. over 3 months post‐SDM diagnosis), target (primary lesion/metastatic lesion/both), systemic therapy regimen (chemotherapy; targeted therapy, including EGFR‐TKIs [gefitinib, erlotinib, osimertinib], ALK inhibitors [crizotinib, alectinib], and other driver gene‐targeted agents based on mutation status). Local treatment was defined as radical local interventions targeting the primary tumor and/or solitary metastatic lesion. Specific modalities included (1) surgical resection: including resections performed for the primary tumor and/or metastatic lesions and (2) radiotherapy: including stereotactic body radiotherapy (SBRT) performed for the primary tumor and/or metastatic lesions, as well as stereotactic radiosurgery (SRS) performed for brain metastases.
  Patient follow‐up was conducted through electronic medical records and telephone interviews. To minimize information bias, all imaging results and disease progression events were independently evaluated by two experienced oncologists.
4. Outcome measures: Time to multiple metastases (TTM: interval from SDM diagnosis to first detection of ≥ 2 new metastases or increased lesion count via imaging); multiple metastases status (imaging‐confirmed ≥ 2 organs or ≥ 2 lesions in one organ during follow‐up). PFS: The interval from the date of SDM diagnosis to the first occurrence of tumor progression (such as multiple metastases, original lesion enlargement exceeding criteria) or death.

### 5.5 Data Analysis

#### 5.5.1 Prognostic Factor Analysis [18]

1. Univariate screening: Cox proportional hazards model calculated HR and 95% CI. In the Cox regression model for multiple metastases‐free survival time, variables with *p* < 0.2 were selected for multivariate analysis, while in the model for PFS, variables with *p* < 0.05 were selected for multivariate analysis.
2. Multivariate modeling: Forward selection built multivariate Cox models incorporating univariate‐screened variables and clinically significant indicators; proportional hazards assumption was validated via Schoenfeld residuals (*p* > 0.05). In the multivariate regression model, reference groups were defined as follows: age ≤60 years, wild‐type for driver genes, N0–1 for N stage, bone for metastatic site, and no local treatment for treatment strategy. The vertical dashed line at HR = 1 implies no association with multiple metastases risk.
3. Subgroup analysis: Kaplan–Meier curves for multiple metastases‐free survival were plotted by local treatment timing (with the 3‐month cut‐off value), with group differences compared via log‐rank test.

#### 5.5.2 Exploration of the Optimal Cut‐Off Point for Local Treatment Timing

Using data‐driven distribution feature analysis and statistical inference models, we systematically explored the optimal cut‐off point for local treatment timing. The distribution characteristics of local treatment timing were displayed through histograms combined with kernel density curves [19]. The MSRS method was used to identify the cut‐off that provides the most significant split in multiple metastases‐free survival. In this analysis, a higher standardized log‐rank statistic indicates a stronger ability of the cut‐off point to distinguish survival differences. This data‐driven approach evaluates all possible time points using log‐rank tests to identify the cut‐off that provides the most significant split in multiple metastases‐free survival. Importantly, it applies rigorous statistical penalization to adjust for the inflation of Type I error caused by multiple testing. This analysis was performed using the maxstat package in R software.

#### 5.5.3 Analysis of RMST and Derived Indicators

To define a potential temporal cut‐off point between “true oligo‐metastasis” and “transitional metastasis,” we employed restricted mean survival time (RMST) and its derived indicators. RMST is defined as the mean duration free of multiple metastases from baseline (time 0) to a specified follow‐up time point (*τ*), corresponding to the area under the Kaplan–Meier curve. Furthermore, the RMST/*τ* ratio was calculated to reflect the average proportion of time patients maintained a metastasis‐free state within the observation period. To detect the presence of a “plateau phase” in the survival curve—which would indicate a stabilization of metastasis risk—we calculated the rate of change in RMST between adjacent time points (ΔRMST/Δ*τ*). If an absolute temporal cut‐off point existed (i.e., entering a plateau), this rate of change would approach zero.

#### 5.5.4 Statistical Software and Tools

SPSS 26.0 was used to perform Cox proportional hazards regression, Kaplan–Meier survival analysis, and log‐rank tests [20]. Rstudio was utilized to calculate the RMST and its derived indicators (RMST/*τ* ratio, ΔRMST/Δ*τ* rate of change), plot trend graphs, explore the optimal cut‐off point for local treatment timing, and visualize the distribution characteristics of local treatment timing and the changing curves of statistics. All statistical tests were two‐sided with a significance level set at *α* = 0.05.Patients with missing key baseline or follow‐up data were excluded from the study, and no data imputation methods were applied. Statistical power analysis was performed using R software based on the Schoenfeld formula for Cox proportional hazards regression (two‐sided *α* = 0.05).

## Author Contributions

**Kang Miao**: conceived and designed the study, analyzed and interpreted data, drafted the manuscript. **Siyu Lei**: conceived and designed the study, analyzed and interpreted data, drafted the manuscript. **Xiaobin Zheng**: collected patient data, assisted with statistical analysis. **Shanshan Yang**: collected patient data, assisted with statistical analysis. **Yujin Li**: collected patient data, assisted with statistical analysis. **Sihui Chen**: collected patient data, assisted with statistical analysis. **Qian Miao**: collected patient data, assisted with statistical analysis. **Longfeng Zhang**: collected patient data, assisted with statistical analysis. **Zihua Zou**: collected patient data, assisted with statistical analysis. **Haipeng Xu**: supervised the study design and data interpretation, revised the manuscript critically for intellectual content, finalized the submission. **Gen Lin**: supervised the study design and data interpretation, revised the manuscript critically for intellectual content, finalized the submission. All authors have read and approved the final manuscript.

This work was supported by the Talent Training Fund of Fujian Cancer Hospital (Grant number: F2427R‐YNY03), the Fujian Provincial Science and Technology Innovation Joint Fund Project funded by the Department of Science and Technology of Fujian Province (Grant number: 2024Y9641), and the National Natural Science Foundation of China (Grant number: 82372954).

## Ethics Statement

This study was approved by the Institutional Ethics Committees of Fujian Cancer Hospital (approval number: K2026‐035‐01) and Beijing Chest Hospital (approval number: LW‐2026‐015). The study was performed in line with the principles of the Declaration of Helsinki.

## Conflicts of Interest

The authors declare no conflicts of interest.

## Supporting information




**Supporting File**: mco270904‐sup‐0001‐SuppMat.pdf.

## Data Availability

The data that support the findings of this study are available from the corresponding author upon reasonable request.
