# Supplementary material for: Early Local Treatment Delays Multiple Metastases in Non‐Small Cell Lung Cancer With Solitary Distant Metastasis: A Multicenter Retrospective Cohort Study
Source: MedComm (2020). 2026 Sep 2;7(9):e70904. doi: 10.1002/mco2.70904 (PMC13537008; doi:10.1002/mco2.70904)
Supplement: Supplementary file 1 [file MCO2-7-e70904-s001.pdf]

Early Local Treatment Delays Multiple Metastases in Non-Small Cell Lung Cancer With Solitary Distant Metastasis: A Multicenter Retrospective Cohort Study

Kang Miao#[1], Siyu Lei#[2], Xiaobin Zheng[2], Shanshan Yang[1], Yujing Li[1], Sihui Chen[1], Qian Miao[1], Longfeng Zhang[1], Zihua Zou[1], Haipeng Xu*[1], Gen Lin*[2]

#These authors contributed equally to this work.

[1]Department of Thoracic Oncology, Clinical Oncology School of Fujian Medical University, NHC Key Laboratory of Cancer and Metabolism, Fujian Cancer Hospital, Fuzhou, China.
[2]Department of Medical Oncology, Beijing Tuberculosis and Thoracic Tumor Research Institute, Beijing Chest Hospital, Capital Medical University, Beijing, China.

Corresponding author:
Gen Lin (lingen@bjxkyy.cn), Haipeng Xu (penghaixu@163.com)

Supplementary Table

Table S1. Subgroup Analysis of TTM by Local Treatment Modality

Local Treatment Modality	No. of Patients	Median TTM (months)	HR	P
**Radiotherapy**	47	35.4	Reference	
**Surgical Resection**	9	33.8	1.21 (0.48–3.02)	0.689
**Other/Combined**	5	NR	—	—
